# A guide for a student-led doctoral-level qualitative methods short course in epidemiology: faculty and student perspectives

**DOI:** 10.1093/ije/dyae029

**Published:** 2024-02-22

**Authors:** Felix Gille, Anja Frei, Marco Kaufmann, Anja Lehmann, Javier Muñoz Laguna, Kimon Papadopoulos, Angela Spörri, Mina Stanikić, Martin Tušl, Federica Zavattaro, Milo Alan Puhan

**Affiliations:** Institute for Implementation Science in Health Care (IfIS), University of Zurich, Zurich, Switzerland; Digital Society Initiative (DSI), University of Zurich, Zurich, Switzerland; Epidemiology, Biostatistics and Prevention Institute (EBPI), University of Zurich, Zurich, Switzerland; Epidemiology, Biostatistics and Prevention Institute (EBPI), University of Zurich, Zurich, Switzerland; Epidemiology, Biostatistics and Prevention Institute (EBPI), University of Zurich, Zurich, Switzerland; Epidemiology, Biostatistics and Prevention Institute (EBPI), University of Zurich, Zurich, Switzerland; EBPI-UWZH Musculoskeletal Epidemiology Research Group, University of Zurich and Balgrist University Hospital, Zurich, Switzerland; University Spine Centre Zurich (UWZH), Balgrist University Hospital and University of Zurich, Zurich, Switzerland; Institute for Implementation Science in Health Care (IfIS), University of Zurich, Zurich, Switzerland; Digital Society Initiative (DSI), University of Zurich, Zurich, Switzerland; Central Informatics, Multimedia an E-Learning Services, University of Zurich, Zurich, Switzerland; Institute for Implementation Science in Health Care (IfIS), University of Zurich, Zurich, Switzerland; Epidemiology, Biostatistics and Prevention Institute (EBPI), University of Zurich, Zurich, Switzerland; Epidemiology, Biostatistics and Prevention Institute (EBPI), University of Zurich, Zurich, Switzerland; Institute for Implementation Science in Health Care (IfIS), University of Zurich, Zurich, Switzerland; Digital Society Initiative (DSI), University of Zurich, Zurich, Switzerland; Epidemiology, Biostatistics and Prevention Institute (EBPI), University of Zurich, Zurich, Switzerland

**Keywords:** Qualitative methods, education, course development, epidemiology, academic training, mixed-methods

## Abstract

Qualitative research and mixed methods are core competencies for epidemiologists. In response to the shortage of guidance on graduate course development, we wrote a course development guide aimed at faculty and students designing similar courses in epidemiology curricula. The guide combines established educational theory with faculty and student experiences from a recent introductory course for epidemiology and biostatistics doctoral students at the University of Zurich and Swiss Federal Institute of Technology, Zurich. We propose a student-centred course with inverse classroom teaching and practice exercises with faculty input. Integration of student input during the course development process helps align the course syllabus with student needs. The proposed course comprises six sessions that cover learning outcomes in comprehension, knowledge, application, analysis, synthesis and evaluation. Following an introductory session, the students engage in face-to-face interviews, focus group interviews, observational methods, analysis and how qualitative and quantitative methods are integrated in mixed methods. Furthermore, the course covers interviewer safety, research ethics, quality in qualitative research and a practice session focused on the use of interview hardware, including video and audio recorders. The student-led teaching characteristic of the course allows for an immersive and reflective teaching-learning environment. After implementation of the course and learning from faculty and student perspectives, we propose these additional foci: a student project to apply learned knowledge to a case study; integration in mixed-methods; and providing faculty a larger space to cover theory and field anecdotes.

Key Messages

- Qualitative research methods and mixed methods are core competencies for epidemiologists.
- Students appreciate a student-centred course design with inverse classroom teaching and practice exercises.
- Students value field anecdotes and explanatory stories from experienced faculty.
- Students particularly value course content related to critical thinking and skills required during interviews.

## Introduction

A recent international effort reaffirmed the view of the Centers for Disease Control and Prevention and European Centre for Disease Prevention and Control that knowledge and application of qualitative research methods are core competencies for epidemiologists.^1–3^ However, they are rarely included in curricula for epidemiologists. Anchored in the social sciences, qualitative methods cover various interview methods, observations and document analysis. The findings derived from qualitative research can inform the design of epidemiological studies, both observational and interventional. Epidemiologists employ qualitative methods to understand social phenomena, values and concepts. Namely, qualitative data can help uncover complexities that might not be explainable with quantitative methods, such as inner experiences and perceptions. The fundamental understanding of population health and its determinants benefits from the conversational integration of qualitative and quantitative approaches in the distinct mixed-methods (MM) research designs.^4^ It is important to recognize that qualitative research methods should not be confused with MM research. The integrated use of qualitative and quantitative research methods produces different kinds of data and results that together provide a fuller picture of the populations studied in epidemiology.^5^ Considering the importance of qualitative research and MM in the field, we argue that early-career training in these methods is essential.^6^

Little guidance exists, aside from Pfadenhauer *et al.* 2018 course development guidance.^7^ Therefore, we—faculty staff and doctoral students—provide a jointly written how-to-do guide for a short course that focuses primarily on qualitative research methods and their connection with MM. The course is designed specifically for doctoral epidemiology students. This guide is rooted in education theory and collective learning and teaching experiences during a 30-h seminar, held as part of the PhD programme in Epidemiology & Biostatistics at the Life Science Graduate School of the University of Zurich and Swiss Federal Institute of Technology in Zurich.^8^

To develop the guidance, all course students were invited to participate in the process. Considering the formal course evaluation, a sub-group of students J.M.L., K.P., M.S., M.T. and F.Z., together with staff member F.G., discussed the course experiences and derived preliminary guidance. Faculty staff A.F., A.L., M.K., A.S., M.A.P., involved in teaching and course administration, provided input. J.M.L., K.P., M.S., M.T. and F.Z. wrote the sessions description to strengthen the students’ perspective in this article.

## How to develop a qualitative and mixed-methods course? — a faculty staff perspective

We suggest following a course development guide covering: expected learning outcomes; teaching approaches; learning material and practice training; and assessment.^9^ During the development process, student engagement helps to anticipate expectations and tailor the course content towards the students’ needs. We defined 14 learning outcomes, see Table 1.^10^

**Table 1.** Learning outcomes

**Knowledge and comprehension**	
1.	Describe MM^a^
2.	Describe qualitative methods
3.	Describe why qualitative methods are useful for epidemiological studies
4.	Identify qualitative and MM studies
5.	Infer knowledge from qualitative and MM studies
6.	Review qualitative studies
7.	Use qualitative research terminology
**Application and analysis**	
8.	Analyse results from qualitative research
9.	Design a qualitative study
10.	Design an MM study
11.	Employ qualitative and MM
**Synthesis and evaluation**	
12.	Evaluate qualitative studies as part of review studies of qualitative research
13.	Recognize errors in qualitative research
14.	Synthesize knowledge from qualitative research

a Mixed methods.

We designed the in-person course as a flipped classroom. The flipped classroom model requires students to take the lead before, during and after the classes.^11^ Alternative student-led and practice-oriented learning approaches are problem-based learning or project-based learning.

Small student groups prepared and taught individual sessions, seeking guidance from faculty members. Concluding each session, faculty members presented a summary and key take-aways. This approach enabled students to delve into specific areas of qualitative and MM research but integrated the course assessment as part of the students’ session performance.

Guided by the course objectives, we designed six sessions (see Table 2) and suggest additional sessions in future courses as outlined in the discussion below. For each session we selected core reading materials (for more detail, see Supplementary Table S1, available as Supplementary data at *IJE* online) while encouraging students to introduce additional teaching materials including videos, blogs or podcasts. We allocated learning material on research ethics as a self-study component, considering that research ethics is an integral part of doctoral education.

**Table 2.** Course overview

Session	Topic
1	Group session: what is qualitative research?
2	Group session: how to conduct face-to-face interviews
3	Group session: practise training with interview hardware
4	Group session: how to conduct focus group interviews and observation
5	Group session: how to analyse qualitative data
6	Group session: what is mixed-methods research?

## Sessions’ content — a student perspective

### Session 1: what is qualitative research

Although quantitative methods are commonly taught in epidemiology, qualitative skills are often neglected. We introduced students to the basics of qualitative research by describing characteristics of qualitative research, how it differs from quantitative approaches and for what purposes qualitative methods are useful. Additionally, we provided an overview of the philosophical foundations of qualitative research, its methodology, and methods. The session activity stimulated ideas and discussions about the advantages and limitations of qualitative research. We asked the students: ‘Why do mothers choose to vaccinate their newborns?’.^7^^,^^12^ Students were divided into two groups, one group discussed how qualitative methods could address this question and the other group discussed how quantitative methods could be used. Both groups jointly discussed their thoughts.

### Session 2: how to conduct face-to-face interviews

Many epidemiological research questions indirectly or directly rely on qualitative input of various stakeholders such as study participants, health professionals or citizens. An important method to collect qualitative data is the face-to-face interview format. We defined qualitative health interviews and introduced structured, semi-structured, and in-depth interviews. With a focus on interviewer safety, we presented different interactional challenges that can arise within the context of distressful health care settings, featuring considerations of involvement, detachment and personal responsibility.^13^^,^^14^ We outlined the mental health risks for researchers and interviewers in emotionally involved interviews.^15^ In the applied part of the session, we ran an exercise in which participants could reflect on the strengths and limitations of two qualitative interview studies. The first study explored misunderstandings associated with prescribing decisions in general practice settings.^16^ The second study focused on children’s perceptions of the mother’s breast cancer and its initial treatment.^17^ Both studies sparked a dynamic discussion about participant selection, study setting and data collection considerations in qualitative studies involving interviews. The analyses and findings of these two manuscripts allowed students to elaborate on the derivation of themes from a data code, different software to manage interview data, and the consistency between data and findings.

### Session 3: how to use interview hardware

Mastering interview hardware is essential for a successful interview. Before the lecture, we had a training session in cooperation with the Multimedia and E-Learning Services Department of the University of Zurich to familiarize ourselves with cameras, microphones, tripods and dictaphones. In the class we introduced practical aspects to consider when planning qualitative interviews, such as the importance of a quiet setting, the presence of natural light and the optimal positioning of cameras and microphones. Considering that many interviews take place in the online environment by using video conference software, we discussed hardware aspects, advantages (more convenient conditions for participants, higher participation and self-authenticity) and disadvantages (non-verbal and social cues, the quality of the interview depends on the technology used and the digital literacy of participants and interviewers) of online interviews.^18^ In collaboration with experts from the Multimedia and E-Learning Services Department, all students conducted a training by using cameras, microphones, audio recorders and tripods, to set up an interview setting and to simulate interviews. Students learned to assemble the equipment and apply techniques from the previous sessions.

### Session 4: how to conduct focus group interviews and observations

Building on the knowledge gained in Session 3, we discussed focus group and observational methods. These have specifically been selected in the context of population-level research as well as stakeholder engagement. We distinguished focus group interviews from face-to-face interviews. We elaborated on the steps necessary to successfully conduct a focus group interview. In addition, we presented good moderator and research practices, and a strategy guide for question-forming.^19^ Afterwards the class was divided into two groups and each was given a research topic to use in a focus group in class with other students: ‘what are the public attitudes regarding genetic modification on human fetuses?’ and ‘what are the public attitudes regarding physician-assisted suicide?’. Both groups determined key areas of discussion from their topic and prepared an introduction, an ice breaker, guiding questions, transition questions and ending questions. Each group conducted a short focus group interview, with two students acting as moderators. A group discussion closed the session with students deliberating on how their focus group went and what lessons and skills they learned from conducting a practice focus group. Finally, we watched a video of an interview with the aim to discuss interview data that go beyond participants’ testimonies, such as facial expressions, body language and the pace of speech.

### Session 5: how to analyse qualitative data

Considering that this course was the first exposure to qualitative analysis for most students, including the presenters of this session, we explained data analysis in a simple way. We emphasized the goal of ‘putting data into categories for later analysis’. Coding and grouping of the data into multilevel categories and themes were explained.^20^ Due to time constraints, no coding exercises were conducted. Four broad approaches to data analysis were introduced, with the focus on inductive and deductive analyses. Thematic analysis, grounded theory, interpretive phenomenological analysis and framework approach, were then discussed in greater detail. It is important to consider that in the analysis process, themes are unpacked to understand how the themes differ by risk, demographic characteristics or other study variables.^21^ The discussion was followed by a group exercise in which we read designated publications and discussed the type of analysis conducted and the presentation of results. This enabled students to gain a better understanding of the different analysis approaches, as well as to see how results presentation varies from one approach to the other. We then discussed quality assessment in qualitative research and how it differs from the concepts used in quantitative research. Reflexivity, triangulation and fair dealing were among the discussed concepts for validity improvement. A wide range of methods exists that is helpful to reach validity in MM research.^22^ We concluded the session by highlighting quality assessment in qualitative research and reporting checklists.^23^^,^^24^

### Session 6: what is mixed-methods research?

In epidemiology, MM are frequently used but not always recognized as such, or at least not explicitly highlighted. To address this issue, we introduced the design, analysis and data integration of mixed-methods research. Oftentimes researchers need to modify or construct an appropriate MM research design based on existing research methods.^25^ When the aim is causal, researchers may also need to triangulate evidence and adopt pluralistic perspectives—although this is complex.^26^ Sequential explanatory designs (i.e. one method is completed before the other) and convergent parallel design (i.e. both methods applied in parallel) were presented and exemplified by four publications.^27–30^ During the session exercise, the students worked in two groups discussing: (i) what is the burden of living with HIV among men who have sex with men in a European country? And (ii) what are barriers that limit access to health care services for undocumented migrants in the Canton of Zurich? Students developed the justification for using an MM approach for the respective research question, the design in terms of the purpose and sequence of methods, the sampling and data collection, the integration of the data/presentation of results and limitations of the chosen methods. The second part of the session introduced the analysis of MM and integration of results. It focused on the importance of explicitly reporting when and how the integration of the qualitative and quantitative data and results was made. An example of a triangulation matrix was presented and exemplified by studies reviewing methods, findings and relationships. Last, challenges of mixed-methods research were discussed.

## Discussion: a student and faculty staff perspective

We aimed to guide through designing and conducting a qualitative methods course that links qualitative research methods with MM for graduate students in epidemiology. This guide follows a student-centred approach and incorporates both student and faculty staff experiences. Qualitative and MM research is best learned with practice sessions, and course content should address doctoral students’ needs. Hence, the content above may require adaptation prior to implementation in alternative academic settings. The course syllabus presented herein is adaptable and can change at the margins of the topic area. Formal course evaluation and faculty and student feedback showed that students particularly valued the course content related to critical thinking and skills required during interviews. Students appreciated the collaborative and relaxed learning environment, as well as the close guidance from faculty during session preparation. They also highlighted the development of transferable skills, including public speaking and synthesis of complex ideas. Some students suggested more practice sessions in relation to the analysis of qualitative data and tailoring the sessions to students’ doctoral research. More research anecdotes and narrative sharing of research experiences by faculty were also suggested. Additional sessions or an in-depth follow-up course on MM research can provide enriched and deepened knowledge.

An inherent limitation of the proposed student-led qualitative and mixed-methods course was the limited experiences of students leading sessions—resulting in varying quality. Future courses should creatively consider a deeper alignment between students and faculty during the session preparation phase to mitigate this limitation.

Overall, after incorporating evaluations, we suggest the following considerations for future courses:

- Course length: six sessions are short and provide a first glimpse of what qualitative and MM are. Eight to ten sessions are a more comprehensive format that allows time for practice sessions, applying the learned content in individual student’s doctoral research and for deepening knowledge in MM research.
- Integration in MM: the integration of qualitative and quantitative methods in MM research is critical to the successful design of MM studies. MM may also contribute to a more pluralistic understanding of causation—a core epidemiological concept. A practice-based learning session dedicated to designing an MM study allows students to engage with integration. An alternative is an advanced follow-up course on MM.^4^
- Student project: as students like to apply the knowledge learned during the course in their ongoing doctoral research, an alternative way of assessment is the addition of a student project task. Without changing the style of teaching, a student project that aligns with or is part of their doctoral research project would allow students to directly apply the gained knowledge in their own research.
- Research experience: students emphasized that they appreciated learning from anecdotes and stories from experienced faculty. Whereas it is important to provide background knowledge, this point emphasizes that qualitative research and MM are very applied fields. Yet to maximize learning, it is important to find the optimal balance between sharing experiences and providing theoretical background knowledge to doctoral students.

## Conclusion

Qualitative and MM research skills will remain a core competency for epidemiologists. Universities have the opportunity to offer appropriate and student-centred education that aligns with doctoral students’ needs and expectations. Future course development experience can add to our proposed and dynamic guidance and will support others in developing qualitative and MM courses. The effort to improve the quality of epidemiology doctoral education depends on open sharing of experiences, cross-fertilization of students and faculty, and collective learning.

## Ethics approval

Ethics approval was not needed as the article does not present a research study. All interview questions asked during the sessions were for training purposes only and no data were collected.

## Supplementary Material

dyae029_Supplementary_Data

## Data Availability

Not applicable.
