# Supplementary material for: A guide for a student-led doctoral-level qualitative methods short course in epidemiology: faculty and student perspectives
Source: Int J Epidemiol. 2024 Feb 22;53(2):dyae029. doi: 10.1093/ije/dyae029 (PMC10883707; doi:10.1093/ije/dyae029)
Supplement: dyae029_Supplementary_Data [file dyae029_supplementary_data.docx]

Supplementary data

Course book: Pope C, Mays N, editors. Qualitative research in health care. Fourth edition. Hoboken: Wiley-Blackwell; 2020

Additional course reading: Wolff B, Mahoney F, Lohiniva AL, Corkum M. Collecting and Analyzing Qualitative Data. In: The CDC Field Epidemiology Manual [Internet]. Oxford University Press; 2019 [cited 2023 Jun 28]. p. 213–28. Available from: <https://academic.oup.com/book/34988/chapter/298664479>

**Table S1: Course Literature**

| Session | Topic | Book Chapter | Literature |
| --- | --- | --- | --- |
| 1 | Group Session: What is qualitative research. | 1-2 | Greenhalgh T, Taylor R. How to read a paper: Papers that go beyond numbers (qualitative research) BMJ 1997; 315 :740 doi:10.1136/bmj.315.7110.740  Giddings, L. S., & Grant, B. M. (2006). Mixed methods research for the novice researcher. *Contemporary nurse*, *23*(1), 3–11. <https://doi.org/10.5172/conu.2006.23.1.3>    Dubé E, Vivion M, Sauvageau C, Gagneur A, Gagnon R, Guay M. “Nature Does Things Well, Why Should We Interfere?”: Vaccine Hesitancy Among Mothers. Qualitative Health Research. 2016;26(3):411-425. doi:10.1177/1049732315573207 |
| Self-study | Ethical Issues self-study. | 3 | Reid, AM., Brown, J.M., Smith, J.M. *et al.* Ethical dilemmas and reflexivity in qualitative research. *Perspect Med Educ* **7,** 69–75 (2018). <https://doi.org/10.1007/s40037-018-0412-2> |
| 2 | Group Session:  How to conduct face-to-face interviews. | 4 | Morse JM. How Different is Qualitative Health Research From Qualitative Research? Do We Have a Subdiscipline? Qualitative Health Research. 2010;20(11):1459-1464. doi:10.1177/1049732310379116 |
| 3 | Group Session: Practice training - How to use interview hardware. | | |
| 4 | Group Session: How to conduct focus group interviews and observations. | 5, 6 | Strassmann, A., Guler, M., Steurer-Stey, C., Lana, K. D., Carron, T., Braun, J., Giroud, P., Peytremann-Bridevaux, I., Puhan, M. A., & Frei, A. (2022). Nationwide implementation of the self-management program "Living well with COPD": Process and effectiveness evaluation using a mixed-methods approach. *Patient education and counseling*, *105*(3), 670–678. <https://doi.org/10.1016/j.pec.2021.06.018> |
| 5 | Group Session: How to analyse qualitative data. | 9,10, 15 | Schmidt C. The Analysis of Semi-Structured Interviews. In: Flick U, von Kardorff E, Steinke I, Jenner B, Hrsg. A Companion to Qualitative Research. SAGE Publications; 2004. S.253-258. |
| 6 | Group Session: What is mixed methods research? | 12 | A.I. Lehmann, G.F. Bauer & R. Brauchli (2022) Intervention effects for direct and indirect participants in an organisational health intervention: A mixed-methods study, Work & Stress, DOI: 10.1080/02678373.2022.2080774    Cerini T, Kunz R, Dalla Lana K, Radtke T, Polhemus A, Puhan MA and Frei A (2021) Evaluation of the Implementation of a Home-Based Exercise Training Program for People With COPD: A Mixed-Methods Study. Front. Rehabilit. Sci. 2:743588. doi: 10.3389/fresc.2021.743588    Hesse-Biber S. Mixed Methods Research: The “Thing-ness” Problem. *Qualitative Health Research*. 2015;25(6):775-788. doi:[10.1177/1049732315580558](https://doi.org/10.1177/1049732315580558) |
